# P-797. Novel Solution to Increase GI Screening in Patients with Streptococcus gallolyticus Bacteremia

**DOI:** 10.1093/ofid/ofaf695.1007

**Published:** 2026-01-11

**Authors:** Bayan M Alghafli, Meaghan Rettele, Nicholas Bennett, Laura Aragon, Philip Adam, Alexander Braun, Justina Gooden, Sarah E Boyd

**Affiliations:** Saint Luke’s (West Region), BJC Health System, Kansas City, Missouri; Saint Luke’s (West Region), BJC Health System, Kansas City, Missouri; Saint Luke's Health System, Kansas City, Missouri; Saint Luke's Health System, Kansas City, Missouri; Saint Luke’s (West Region), BJC Health System, Kansas City, Missouri; Saint Luke’s (West Region), BJC Health System, Kansas City, Missouri; Saint Luke’s (West Region), BJC Health System, Kansas City, Missouri; Saint Luke's Health System, Kansas City, Missouri

## Abstract

**Background:**

*Streptococcus gallolyticus* bacteremia is linked to colorectal cancer (CRC). Despite its high correlation, many patients do not receive CRC screening. We present our multi-phasic care improvement project evaluating care gaps in CRC screening and subsequent implementation of a templated microbiology comment to improve clinical decision-making for patients with *S. gallolyticus* bacteremia.

**Methods:**

Phase 1 was a retrospective, multi-site cohort study of adults admitted to Saint Luke’s Health System from January 1, 2015 to April 30, 2023 with *S. gallolyticus* isolated in ≥1 blood cultures. The primary endpoint was documented CRC evaluation via colonoscopy within 6 months of the first positive culture. Secondary endpoints included infectious disease (ID) or gastroenterology (GI) consults, diagnostic imaging (e.g., computed tomography abdomen/pelvis [CTAP], echocardiogram [ECHO]), and documented CRC screening recommendations. Phase 2 involved evaluation and deployment of a templated comment, stating: “*Streptococcus gallolyticus* (formerly *S. bovis*) bacteremia may warrant further GI evaluation” on the culture result to nudge CRC screening. Phase 3 was a post-intervention assessment of patients with *S. gallolyticus* bacteremia admitted between Feb 1, 2024 and December 31, 2024 with similar outcomes as in Phase 1.

**Results:**

In Phase 1, 38 patients met inclusion criteria. CRC evaluation was recommended in 65.7% of cases and colonoscopy completed in 34.2%. ID and GI were consulted in 76.3% and 44.7% of patients, respectively. CTAP and ECHO were completed during admission in 47.4% and 81.6% of cases. In Phase 3, 6 patients met criteria with CRC evaluation recommended in 100% and completed in 33%. ID and GI consults occurred in 83.3% and 50% of cases while CTAP and ECHO were completed in 66.7% and 100%, respectively.

**Conclusion:**

Embedding a comment within microbiology results appears to be a high-impact strategy to enhance CRC screening in patients with *S. gallolyticus* bacteremia. Our findings emphasize gaps between recommended and completed screenings, but early post-intervention data suggest improved clinician awareness. Continued evaluation is warranted to determine long-term effectiveness of this intervention.

**Disclosures:**

All Authors: No reported disclosures

